# Serum keratin 19 (CYFRA21-1) links ductular reaction with portal hypertension and outcome of various advanced liver diseases

**DOI:** 10.1186/s12916-020-01784-7

**Published:** 2020-11-12

**Authors:** Karim Hamesch, Nurdan Guldiken, Mahmoud Aly, Norbert Hüser, Daniel Hartmann, Pierre Rufat, Marianne Ziol, Katharina Remih, Georg Lurje, Bernhard Scheiner, Christian Trautwein, Mattias Mandorfer, Thomas Reiberger, Sebastian Mueller, Tony Bruns, Pierre Nahon, Pavel Strnad

**Affiliations:** 1 Medical Clinic III, Gastroenterology, Metabolic Diseases and Intensive Care, University Hospital RWTH Aachen, Pauwelsstr. 30, 52074 Aachen, Germany; 2 Department of Medicine and Infectious Diseases, Faculty of Veterinary Medicine, University of Sadat City, Sadat City, Egypt; 3 Department of Surgery, Technical University of Munich, School of Medicine, Klinikum rechts der Isar, 81675 Munich, Germany; 4 AP-HP, Service d’Biostatistic Hopital Jean Verdier, Bondy, France; 5 Unité de Formation et de Recherche Santé Médecine et Biologie Humaine, Université Paris 13, Communauté d’Universités et Etablissements Sorbonne Paris Cité, Paris, France; 6 Centre de Recherche des Cordeliers, INSERM, Sorbonne Université, USPC, Université Paris Descartes, Université Paris Diderot, F-75006 Paris, France; 7 Centre de ressources biologiques du groupe hospitalier Paris-Seine-Saint-Denis, BB0033-00027, Hôpitaux Universitaires Paris-Seine-Saint-Denis, Assistance Publique Hôpitaux de Paris, Bondy, France; 8 Department of Surgery and Transplantation, University Hospital Aachen, Aachen, Germany; 9 Department of Surgery, Campus Charité Mitte | Campus Virchow-Klinikum-Charité-Universitätsmedizin Berlin, Berlin, Germany; 10 Vienna Hepatic Hemodynamic Lab, Division of Gastroenterology und Hepatology, Department of Internal Medicine III, Medical University Vienna, Vienna, Austria; 11 Salem Medical Center and Center for Alcohol Research, University of Heidelberg, Heidelberg, Germany; 12 Department of Internal Medicine IV, Gastroenterology, Hepatology and Infectious Diseases, Jena University Hospital, Jena, Germany; 13 Integrated Research and Treatment Center, Center for Sepsis Control and Care (CSCC), Jena University Hospital, Jena, Germany; 14 AP-HP, Service d’Hépatologie, Hopital Jean Verdier, Bondy, France; 15 Université Paris 13, Sorbonne Paris Cité, “Equipe labellisée Ligue Contre le Cancer”, F-93206 Saint-Denis, France; 16 Inserm, UMR-1162, “Génomique fonctionnelle des tumeur solides”, F-75000 Paris, France

**Keywords:** Keratin, Biomarker, Chronic liver disease, Cirrhosis, Ductal reaction, Prognostic

## Abstract

**Background:**

Keratins (Ks) represent tissue-specific proteins. K18 is produced in hepatocytes while K19, the most widely used ductular reaction (DR) marker, is found in cholangiocytes and hepatic progenitor cells. K18-based serum fragments are commonly used liver disease predictors, while K19-based serum fragments detected through CYFRA21-1 are established tumor but not liver disease markers yet. Since DR reflects the severity of the underlying liver disease, we systematically evaluated the usefulness of CYFRA21-1 in different liver disease severities and etiologies.

**Methods:**

Hepatic expression of ductular keratins (K7/K19/K23) was analyzed in 57 patients with chronic liver disease (cohort i). Serum CYFRA21-1 levels were measured in 333 Austrians with advanced chronic liver disease (ACLD) of various etiologies undergoing hepatic venous pressure gradient (HVPG) measurement (cohort ii), 231 French patients with alcoholic cirrhosis (cohort iii), and 280 hospitalized Germans with decompensated cirrhosis of various etiologies (cohort iv).

**Results:**

(i) Hepatic K19 levels were comparable among F0–F3 fibrosis stages, but increased in cirrhosis. Hepatic K19 mRNA strongly correlated with the levels of other DR-specific keratins. (ii) In ACLD, increased serum CYFRA21-1 associated with the presence of clinically significant portal hypertension (CSPH; HVPG ≥ 10 mmHg) (OR = 5.87 [2.95–11.68]) and mortality (HR = 3.02 [1.78–5.13]; median follow-up 22 months). (iii) In alcoholic cirrhosis, elevated serum CYFRA21-1 indicated increased risk of death/liver transplantation (HR = 2.59 [1.64–4.09]) and of HCC (HR = 1.74 [1.02–2.96]) over the long term (median follow-up 73 months). (iv) In decompensated cirrhosis, higher serum CYFRA21-1 predicted 90-day mortality (HR = 2.97 [1.92–4.60]) with a moderate accuracy (AUROC 0.64), independently from established prognostic scores.

**Conclusions:**

Hepatic K19 mRNA and serum CYFRA21-1 levels rise in cirrhosis. Increased CYFRA21-1 levels associate with the presence of CSPH and reliably indicate mortality in the short and long term independently of conventional liver biochemistry markers or scoring systems. Hence, the widely available serum CYFRA21-1 constitutes a novel, DR-related marker with prognostic implications in patients with different settings of advanced liver disease.

## Background

Keratins are the intermediate filaments of epithelial cells that are expressed in a tissue-specific manner and therefore constitute widely used biomarkers [1, 2]. Keratins are subdivided into type I proteins (including K1–K8) and type II proteins (including K9–K20), and both types assemble to form heteropolymers. As a consequence, each cell type has a characteristic type I–type II keratin repertoire [3]. For example, keratins 8/18 (K8/K18) are ubiquitously produced in single-layered epithelia while K7/K19 are found in some, but not all, simple epithelial cells [1, 2]. In the liver, K19 constitutes the most widely used histologic marker of ductal/ductular reaction (DR) as it is expressed in cholangiocytes and hepatic progenitor cells (HPC) but not in adult hepatocytes [1, 4].

Upon tissue injury, keratins are released into the serum and therefore are well-known disease markers [5]. K18-based serum markers pose the most prominent keratin biomarkers as they represent useful surrogates of hepatocellular injury [5, 6]. Among them, M30/M65 are among the most widely used severity markers in multiple liver disorders [5]. In contrast, while serum CYFRA21-1 is widely available in routine laboratories as a tumor marker [7, 8], its usefulness in liver disease has not been systematically studied.

The liver possesses two fundamentally different strategies to recover from parenchymal losses, i.e., the division of mature hepatocytes and the progenitor cell-based DR [9, 10]. Division of mature hepatocytes is typically seen after surgical liver resection, but may become impaired during chronic liver diseases, especially at stages with advanced liver scarring (fibrosis). In the latter, DR constitutes the predominant mode of parenchymal replenishment [9, 10]. DR is driven by HPC that can differentiate to both biliary and hepatocellular lineage [11]. Since regeneration typically reflects the rate of parenchymal loss, the extent of DR mirrors the severity of the underlying liver disease [9, 10] and thus may predict the disease course [10, 12].

In line, multiple studies showed that the amount of K7/K19-positive cells correlates with the severity of liver disease and is of prognostic relevance [12–17]. However, these studies only evaluated the amount of DR-specific keratins in tissue sections, which are less commonly available due to the increasing use of non-invasive methods for diagnosing and staging liver fibrosis and portal hypertension. Hence, the aim of this study was to test whether a serum marker reflecting the extent of DR would be a valuable tool for prognostication. Thus, we systematically analyzed hepatic K19 expression in liver biopsies from various chronic liver diseases and assessed the significance of CYFRA21-1 serum levels in three further, well-characterized cohorts of patients with different settings of advanced liver disease.

## Methods

### Analysis of human liver samples

(Cohort i) 57 liver samples from patients with liver disease of different etiologies and 13 control samples were assessed for hepatic expression of the ductular keratins K19, K7, and K23 (Additional file 1: Table S1). Further details on patient selection and their molecular analysis are given in the supplement (Additional file 1).

### Analysis of CYFRA 21-1 levels in patients with different liver disease stages

Three well-characterized cohorts were used to assess serum CYFRA21-1 in relation to liver disease severity and clinical outcomes: (cohort ii; Table 1) 333 Austrians with advanced chronic liver disease (ACLD; as defined by a hepatic venous pressure gradient (HVPG) ≥ 6 mmHg [18]) of various etiologies undergoing HVPG measurement, (cohort iii; Table 2) 231 French patients with alcoholic cirrhosis, and (cohort iv; Table 3) 280 Germans hospitalized for decompensation of cirrhosis. Further details are given in the supplement (Additional file 1).

### Statistical analysis

Continuous variables were given as median with interquartile range (IQR) and were compared by the Mann-Whitney *U* test or Kruskal-Wallis test. Categorical variables were reported as absolute (*n*) and relative (%) frequencies, and contingency tables were analyzed with chi-squared tests.

The optimal cut-off values for CYFRA 21-1 as an indicator of CSPH or survival were determined using a cohort-specific Youden’s index in receiver operating characteristic (ROC) analysis. Areas under the receiver operating characteristic curve (AUROC) were calculated. Univariate and multivariable analyses of risk factors for outcomes such as mortality were conducted using Cox regression analyses to calculate hazard ratios (HRs). Distributions among groups were assessed by univariable logistic regression analyses to calculate odds ratios (ORs), and forward-stepwise multiple logistic regression analyses were used to test for independent associations.

Time-to-event variables were displayed using Kaplan-Meier curves, and differences in event-free survival (or HCC occurrence, respectively) were tested using the log-rank test. For the analysis of transplant-free survival, death or transplant was considered as endpoints and patients were right-censored at the end of the observation period.

HRs and ORs were given with their corresponding 95% confidence intervals (CI) [in brackets].

Correlations between clinical variables and serum biomarkers or elastography parameters were assessed by calculating Spearman’s rank correlation (Spearman’s rho) coefficients.

Nominal *P* values were reported for all statistical tests. Due to the exploratory nature of most parts of our study, we applied two-sided significance levels of *P* < .05 for all tests without correction for multiple testing. Statistical analyses were performed using the SAS System Package version 8.02 (SAS Institute, Cary, NC, USA) and SPSS version 23 (IBM, Armonk, NY, USA), and graphs were created with SPSS or Prism 5 (GraphPad, La Jolla, CA, USA).

## Results

### Hepatic K19 levels are upregulated in patients with liver cirrhosis

**Fig. 1:**
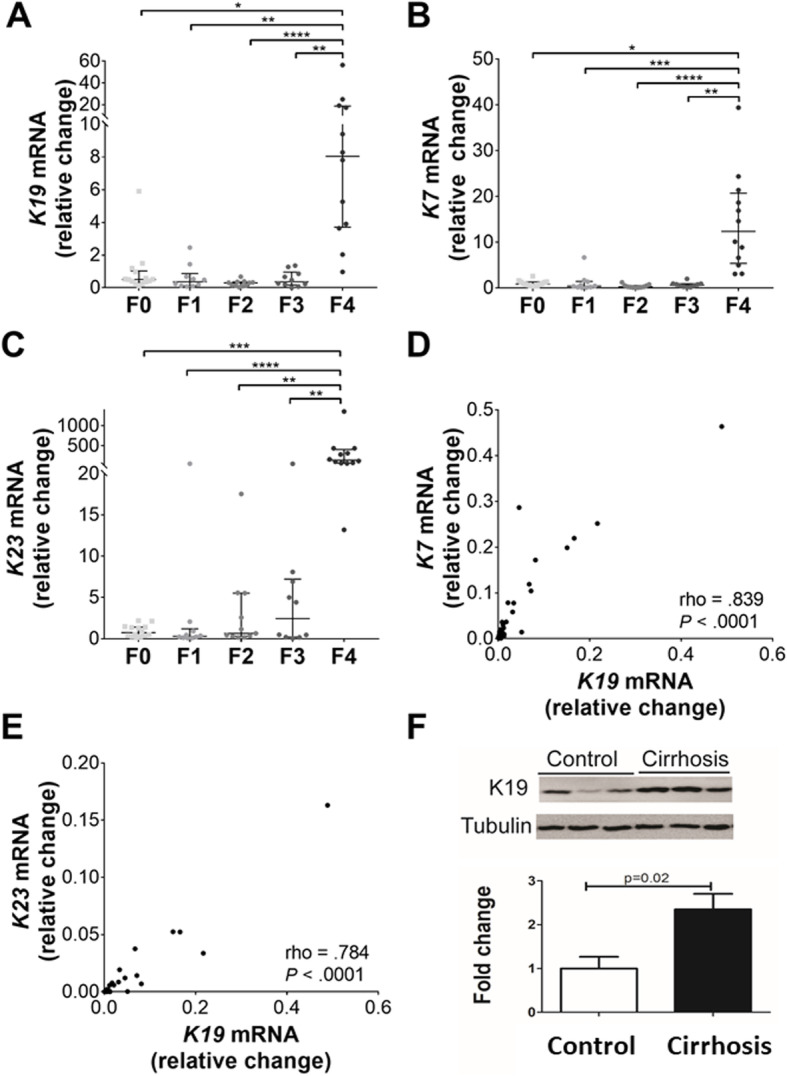
Hepatic expression of genes related to ductular reaction in patients with chronic liver disease (cohort i). Fifty-seven livers from patients with various stages of liver fibrosis (fibrosis stages F0–F4) were analyzed (cohort i). **a**–**c** Relative hepatic expression of keratins 19, 7, and 23 (K19/K7/K23), compared to RPLP0. Average K7/19/23 expression in control subjects was arbitrarily set as 1 and all other levels represent a ratio. **d**, **e** Spearman’s correlation coefficient quantifies the relationship between the hepatic expression of K19 mRNA and K7 (**d**) or K23 (**e**). **f** K19 protein levels were determined by immunoblotting and tubulin was used as a loading control. The relative band intensity (lower panel) was quantified using ImageJ. *P < .05, **P < .01, ***P < .001, ****P < .0001

First, we analyzed the hepatic expression of ductular keratins (K7/19/23) in 57 patients with different liver fibrosis stages and disease etiologies (Additional file 1: Table S1). K19 mRNA levels were comparable among patients without fibrosis (F0) and with mild to advanced fibrosis stages (F1–F3), but elevated in cirrhotic individuals (F4; Fig. 1a). Notably, the increase seemed to be unrelated to the etiology of liver disease, since comparable K19 mRNA levels were seen in samples from patients with NAFLD, ALD, and chronic hepatitis C (Additional file 1: Fig. S1A). Similar results were obtained with the ductular keratins K7/K23 (Fig. 1b, c; Additional file 1: Fig. S1B, C). Moreover, the expression of K7/K19 and K19/K23 strongly correlated with each other (Fig. 1d, e). The mRNA results were confirmed by immunohistochemistry and immunoblotting against K19 revealing a higher number of K19-positive cells and K19 protein levels in cirrhotic vs. control livers (Fig. 1f and Additional file 1: Fig. S2).

Taken together, levels of ductular keratins were elevated in patients with cirrhosis most likely as a consequence of a prominent DR.

### Increased CYFRA21-1 associates with clinically significant portal hypertension (CSPH) and poor survival in patients with advanced chronic liver disease (ACLD)

**Fig. 2:**
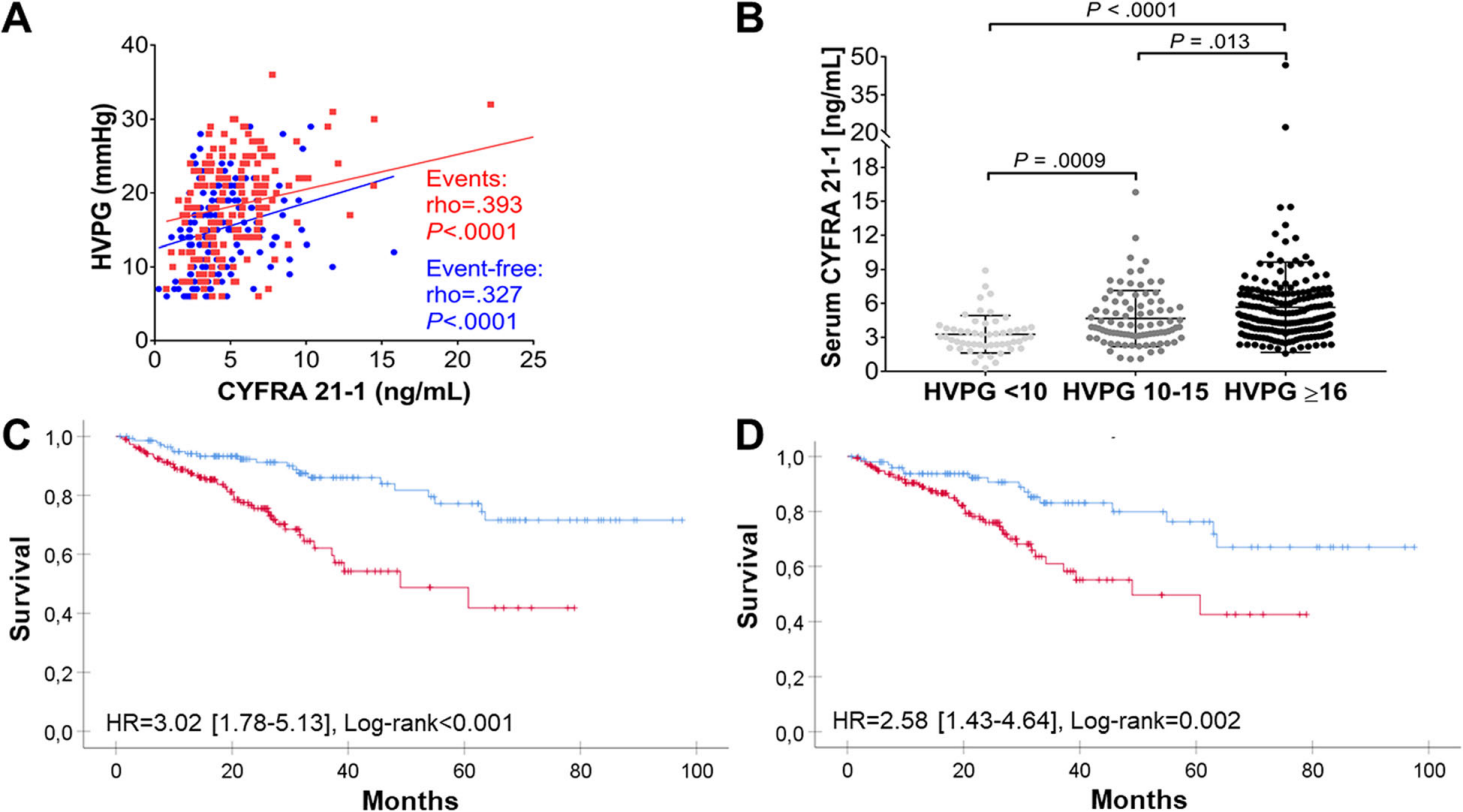
Serum CYFRA21-1 as an indicator of clinically significant portal hypertension (CSPH) and transplant-free liver-related survival in patients with portal hypertension due to advanced chronic liver disease (ACLD; cohort ii). Three hundred thirty-three patients (median follow-up of 22 months) were analyzed (cohort ii). **a** Correlation of serum CYFRA21-1 and hepatic venous pressure gradient (HVPG). Data from patients with hepatic decompensation or liver-related death (events) are depicted in red color whereas the data from patients without hepatic decompensation or liver-related death (event-free) are depicted in blue color. **b** Serum CYFRA21-1 levels in three strata of ACLD patients with HVPG of 6–9 mmHg (no CSPH), 10–15 mmHg, and ≥ 16 mmHg. The serum CYFRA21-1 cut-off of 3.90 ng/mL was used to generate Kaplan-Meier curves (CYFRA21-1 < 3.90 ng/mL in blue, CYFRA21-1 ≥ 3.90 ng/mL in red) for the following outcomes: **c** the cumulative transplant-free, liver-related survival in the overall cohort and **d** the cumulative transplant-free, liver-related survival in patients with CSPH (HVPG ≥ 10 mmHg). Unadjusted hazard ratios (HRs) with their 95% confidence intervals [in brackets] and log-rank *P* values are shown in the corresponding graphs

To further assess the biological meaning and prognostic usefulness of serum CYFRA21-1 levels, we analyzed a thoroughly characterized Austrian cohort of 333 patients with ACLD of different etiologies. Two hundred eighty patients had CSPH (HVPG ≥ 10 mmHg), and these subjects had, as expected, higher Child-Pugh and model for end-stage liver disease (MELD) scores than their 53 counterparts without CSPH (Table 1). Serum CYFRA21-1 levels correlated with HVPG values in the overall cohort (rho = .375, *P* < .001). A similar correlation was seen in patients who experienced hepatic decompensation or liver-related death and those who did not (Fig. 2a). Accordingly, serum CYFRA21-1 was markedly higher in CSPH subjects (Table 1) and increased CYFRA21-1 values strongly associated with CSPH (CYFRA21-1 ≥ 3.90 ng/mL: unadjusted OR = 5.87 [2.95–11.68], *P* < .001). Notably, the association between CYFRA21-1 ≥ 3.90 ng/mL and CSPH remained highly significant after adjustment for etiology and demographic parameters (adjusted OR [aOR] = 5.47 [2.65–11.29], *P* < .001). Similarly robust associations were achieved after adjustment for platelets, as a simple non-invasive marker for portal hypertension, as well as the prognostic composite scores Child-Pugh and MELD (Table 4). An assessment of CYFRA21-1 as a dichotomized and continuous variable revealed comparable results (Table 2). Finally, CYFRA21-1 levels differed significantly between HVPG strata, i.e., ACLD patients without CSPH (i.e., HVPG 6-9 mmHg), HVPG 10–15 mmHg, and ≥ 16 mmHg (Fig. 2b). The AUROC for serum CYFRA21-1 indicating CSPH was 0.75 (0.69–0.82) while the AUROC for the composite score MELD was only slightly higher (0.77 (0.70–0.83)). Combining CYFRA21-1 with MELD yielded a numerically higher AUROC (0.82 (0.76–0.88)) (Additional file 1: Suppl. figure S3).

**Table 1:** Characteristics of the patient cohort with portal hypertension due to advanced chronic liver disease (ACLD), stratified by the presence of clinically significant portal hypertension (CSPH) (cohort ii)

	ACLD (***n*** = 333)	No CSPH (***n*** = 53)	CSPH (***n*** = 280)	***P*** value
Age (years)	54 (15)	55 (14)	54 (15)	0.51
Male sex (*n*, %)	243 (73)	40 (76)	203 (73)	.66
Alcohol (*n*, %)	119 (36)	6 (11)	113 (40)	< .001
Non-alcoholic fatty liver (*n*, %)	28 (8)	5 (9)	23 (8)	
Viral (*n*, %)	141 (42)	38 (72)	103 (37)	
Child-Pugh score (points)	6 (3)	5 (0)	7 (4)	< .001
MELD score (points)	10 (5)	8 (2)	11 (5)	< .001
ALT (U/L)	41 (51)	61 (66)	40 (46)	.001
AST (U/L)	59 (53)	60 (52)	58 (53)	.97
Bilirubin (mg/dL)	1.1 (1.1)	0.6 (0.4)	1.2 (1.3)	< .001
Albumin (g/L)	36.7 (8.3)	40.5 (4.2)	35.7 (7.9)	< .001
INR	1.20 (0.30)	1.10 (0.18)	1.30 (0.22)	< .001
Creatinine (mg/dL)	0.77 (0.24)	0.87 (0.27)	0.74 (0.23)	.001
Platelet count (G/L)	105 (79)	152 (63)	99 (62)	< .001
HVPG (mmHg)	17 (10)	8 (2)	18 (7)	< .001
CYFRA21-1 (ng/mL)	4.3 (3.3)	3.0 (1.5)	4.6 (3.3)	< .001
Liver-related death (*n*, %)	70 (21)	10 (19)	60 (21)	.68
Hepatic decompensation or liver-related death (*n*, %)	176 (53)	19 (36)	157 (56)	.007

Portal hypertension was defined as patients having a hepatic venous pressure gradient (HVPG) ≥ 6 mmHg whereas CSPH was defined as HVPG ≥ 10 mmHg. Quantitative measures are shown as median (interquartile range) or as an absolute count (*n*) and relative frequency (%). *Abbreviations*: *MELD* model of end-stage liver disease, *ALT* alanine aminotransferase, *AST* aspartate aminotransferase, *INR* international normalized ratio

**Table 2:** Binary logistic regression models for clinically significant portal hypertension (CSPH) using dichotomized and continuous variables for serum CYFRA21-1 in Patients with portal hypertension due to ACLD (cohort ii)

	Serum CYFRA21-1, dichotomized ≥ 3.90 ng/mL		Serum CYFRA21-1, per ng/mL increase	
	Odds ratio (95% CI)	***P*** value	Odds ratio (95% CI)	***P*** value
**Unadjusted**	5.87 [2.95–11.68]	< 0.001	1.73 [1.40–2.14]	< 0.001
**Adjusted for age and sex**	6.19 [3.09–12.39]	< 0.001	1.77 [1.42–2.20]	< 0.001
**Adjusted for etiology**	5.25 [2.58–10.70]	< 0.001	1.73 [1.38–2.18]	< 0.001
**Adjusted for age, sex, and etiology**	5.47 [2.65–11.29]	< 0.001	1.79 [1.41–2.28]	< 0.001
**Adjusted for platelet count**	6.74 [3.19–14.22]	< 0.001	1.75 [1.40–2.18]	< 0.001
**Adjusted for MELD score**	4.07 [1.99–8.33]	< 0.001	1.53 [1.23–1.90]	< 0.001

CSPH was defined as hepatic venous pressure gradient (HVPG) ≥ 10 mmHg. CYFRA21-1 was dichotomized using a cut-off determined by the maximum Youden index for the presence of CSPH. *Abbreviations*: *CYFRA21-1* fragments of keratin 19, *MELD* model for end-stage liver disease

In Kaplan-Meier analysis, CYFRA21-1 ≥ 3.90 ng/mL indicated a poor liver-related transplant-free survival over a median follow-up of 22 months (univariate Cox regression analysis: HR = 3.02 [1.78–5.13] log-rank *P* < .001; Fig. 2c). Multivariable Cox regression analyses accounting for relevant confounders confirmed these associations (Additional file 1: Table S2). A similar association with liver-related death was observed when only patients with CSPH were considered (univariate HR = 2.58 [1.43–4.64], log-rank *P* = .002; Fig. 2d).

Collectively, increased serum CYFRA21-1 levels were strongly and independently associated with the presence of CSPH and poor survival in patients with ACLD. Of note, CYFRA21-1 provided prognostic information in the subgroup of patients with CSPH, highlighting its prognostic relevance beyond being a non-invasive surrogate of CSPH.

### CYFRA21-1 indicates poor long-term survival in alcoholic cirrhosis

To validate the long-term prognostic usefulness of serum CYFRA21-1 levels, we turned to a French cohort of 231 patients with alcoholic cirrhosis, who were followed up longitudinally for HCC development (Table 3). During the median follow-up of 73 months, 98 patients died or received a liver transplant (non-survivors), while 133 patients survived without the need for a liver transplant (transplant-free survivors; Table 3). While transplant-free survivors and non-survivors did not differ in their demographic characteristics (Table 3), non-survivors more often developed hepatocellular carcinoma (47.4% vs. 6.8%, *P* < .0001; Table 3). 44.9% of non-survivors reached the pre-defined endpoint due to HCC while 51.0% did so due to other liver-related reasons. The baseline CYFRA21-1 serum level was higher in non-survivors vs. survivors (Table 3). A comparison of patients with high versus low serum CYFRA21-1 using the median (5.26 ng/mL) as a cut-off revealed that patients with higher CYFRA21-1 levels had more often ascites, had higher Child-Pugh scores, and reached the pre-defined endpoint of HCC more frequently (Additional file 1: Table S3).

**Table 3:** Characteristics of the patient cohort with cirrhosis due to long-term alcohol misuse included in HCC surveillance programs, stratified by liver-related survival (cohort iii)

	Alcoholic cirrhosis (***n*** = 231)	Transplant-free survivors (***n*** = 133)	Non-survivors* (***n*** = 98)	***P*** value
Age at cirrhosis diagnosis (years)	56.5 (12.4)	56.4 (10.8)	56.6 (14.9)	.61
Male sex	185 (80.1%)	105 (78.9%)	80 (81.6%)	.64
BMI (kg/m^2^)	27.0 (6.0)	27.0 (5.9)	27.5 (7.0)	.07
Diabetes mellitus	70 (30.4%)	38 (28.6%)	32 (32.7%)	.50
Ascites	87 (37.4%)	45 (33.8%)	42 (42.9%)	.19
Hepatic encephalopathy	23 (10.0%)	9 (6.8%)	14 (14.3%)	.056
Child-Pugh score (points)	6 (3)	6 (3)	7 (3)	< .001
HCC during follow-up	56 (23.9%)	9 (6.8%)	47 (48.0%)	< .001
Survival (years)	5.0 (6.5)	5.0 (7.3)	4.8 (5.0)	.78
Liver transplantation	17 (7.4%)	–	17 (17.5%)	NA
Liver-related death	93 (40.4%)	–	93 (95.9%)	NA
Death	–	–	98 (100%)	NA
*HCC-related*			*44 (44.9%)*	
*Liver-related*			*50 (51.0%)*	
*Extra-hepatic*			*4 (4.1%)*	
ALT (× ULN)	1.0 (1.0)	1.0 (1.0)	1.0 (1.0)	.28
AST (× ULN)	2.0 (1.0)	2.0 (1.0)	2.0 (1.5)	.99
GGT (× ULN)	4.0 (6.0)	4.0 (6.0)	5.0 (6.0)	.45
Bilirubin (μmol/L)	23.0 (35.5)	21.0 (31.0)	33.0 (47.0)	.001
Albumin (mg/dL)	36.3 (10.3)	39.0 (9.4)	34.0 (10.1)	.001
PT (% of control)	65.0 (30.5)	68.0 (27.0)	57.0 (30.5)	< .001
Platelet count (G/L)	123.5 (74)	129.0 (83.0)	114.0 (57.0)	.08
CYFRA21-1 (ng/mL)	5.26 (1.75)	4.99 (1.81)	5.58 (1.75)	.008

Quantitative measures are shown as median (interquartile range) or as an absolute count (*n*) and relative frequency (%). Liver transaminases are displayed as a multiple of the upper limit of normal (ULN). *Abbreviations*: *BMI* body mass index, *HCC* hepatocellular carcinoma, *ALT* alanine aminotransferase, *AST* aspartate aminotransferase, *GGT* gamma-glutamyl transferase, *PT* prothrombin time. *Non-survivors include patients who died or received a liver transplantation within the observation period (median follow-up of 73 months)

**Fig. 3:**
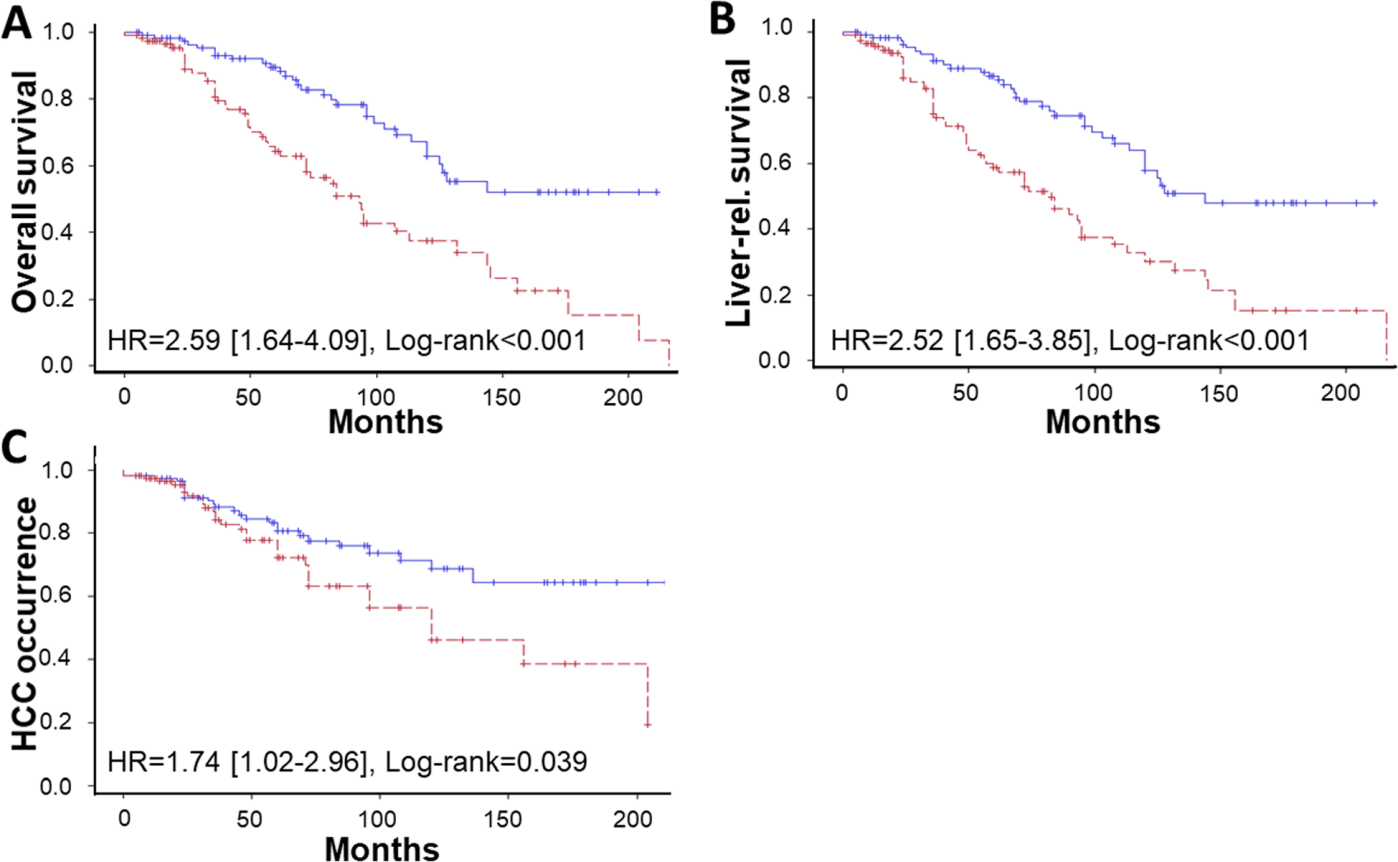
Serum CYFRA21-1 as an indicator of long-term death and HCC occurrence in patients with alcoholic cirrhosis (cohort iii). Two hundred thirty-one patients with cirrhosis due to long-term alcohol misuse (median follow-up of 73 months) were analyzed. The median serum CYFRA21-1 value was used to generate Kaplan-Meier curves (CYFRA21-1 < 5.26 ng/mL in blue, CYFRA21-1 ≥ 5.26 ng/mL in red) for the following outcomes: **a** the overall transplantation-free survival, **b** the liver-related, not HCC-related transplantation-free survival, and **c** the occurrence of HCC. Unadjusted hazard ratios (HRs) with their 95% confidence intervals [in brackets] and log-rank *P* values are shown in the corresponding graphs

In Kaplan-Meier analysis, CYFRA21-1 serum levels ≥ 5.26 ng/mL were associated with reduced cumulative overall transplantation-free survival (univariate HR = 2.59 [1.64–4.09], log-rank *P* < .001; Fig. 3a). Similar results were seen when only liver-related, not HCC-related endpoints were considered (univariate HR = 2.52 [1.65–3.85], *P* < .001; Fig. 3b). Using Cox’s proportional hazards model, higher serum CYFRA21-1 levels (HR per ng/mL increase 1.22 [1.02–1.43], *P* = .02; Additional file 1: Table S4) along with higher age and higher Child-Pugh score were independently associated with overall death/need for transplantation. To further estimate the diagnostic accuracy of serum CYFRA21-1 for indicating mortality, we performed a ROC analysis. The corresponding AUROC was 0.60 (0.53–0.68) (Additional file 1: Suppl. figure S4), while the AUROC for the Child-Pugh score—which also encompasses clinical variables—was 0.63 (0.56–0.71).

Finally, higher CYFRA21-1 serum levels were associated with a somewhat higher occurrence of HCC (univariate HR = 1.74 [1.02–2.96], log-rank *P* = .039; Fig. 3c). The positive predictive value and negative predictive value of the median CYFRA21-1 levels of 5.26 ng/mL for HCC occurrence were 22% and 74%, respectively. Using the same Cox’s proportional hazards model as for mortality, higher serum CYFRA21-1 levels were not significantly associated with HCC occurrence (Additional file 1: Table S4).

Taken together, in patients with alcoholic cirrhosis, serum CYFRA21-1 was an independent indicator of overall and liver-related long-term mortality.

### CYFRA21-1 indicated short-term survival in patients with decompensated cirrhosis and ascites

To determine the predictive ability of serum CYFRA21-1 levels for short-term mortality in decompensated cirrhosis, we analyzed a cohort of patients hospitalized due to hepatic decompensation with ascites (Table 4). Within 90 days of follow-up, 88 patients (31.4%) either died or were transplanted. These “non-survivors” were significantly older, more commonly had HCC or spontaneous bacterial peritonitis, and displayed higher MELD, Child-Pugh, and acute-on-chronic liver failure (ACLF) scores than transplant-free survivors (Table 4). Non-survivors had higher median CYFRA21-1 serum levels than survivors (Table 4). In the entire cohort, CYFRA21-1 only weakly correlated with ALT, AST, and MELD, while it did not significantly correlate with markers of liver synthetic function (e.g., INR and albumin; Additional file 1: Table S5). The CYFRA21-1 serum values were 8.5 ± 12.5 ng/mL in cirrhotic patients without ACLF and 8.2 ± 9.0 ng/mL, 12.4 ± 13.4 ng/mL, and 19.1 ± 14.8 ng/mL in patients with ACLF grades 1, 2, and 3, respectively (Additional file 1: Figure S5).

**Table 4:** Characteristics of hospitalized patients with decompensated cirrhosis and ascites, stratified by liver-related survival (cohort iv)

	Decompensated cirrhosis (***n*** = 280)	Transplant-free survivors* at 90 days (***n*** = 192)	Non-survivors** at 90 days (***n*** = 88)	***P*** value
Age (years)	59 (52–67)	57 (48–64)	63 (55–69)	< 0.001
Male sex	206 (74)	142 (74)	64 (73)	0.88
Alcoholic liver disease	220 (79)	158 (82)	62 (70)	0.03
Self-reported alcohol use within the 30 days before admission	128 (46)	91 (47)	37 (42)	0.44
HCC	39 (14)	21 (11)	18 (20)	0.04
SBP	37 (13)	14 (7)	23 (26)	< 0.0001
Child-Pugh C	174 (62)	109 (57)	65 (74)	< 0.01
MELD score	17 (12–22)	16 (12–20)	22 (14–28)	< 0.000001
ACLF	73 (26)	34 (18)	39 (44)	< 0.00001
ACLF grade (1/2/3)	40 (55)/20 (27)/13 (18)	24 (71)/9 (26)/1 (3)	16 (41)/11 (28)/12 (31)	< 0.01
ALT (μmol L^−1^ s^−1^)	0.56 (0.38–0.88)	0.56 (0.38–0.77)	0.58 (0.41–1.16)	0.07
AST (μmol L^−1^ s^−1^)	1.07 (0.70–1.86)	1.04 (0.68–1.71)	1.12 (0.73–2.55)	0.14
Bilirubin (μmol L^−1^)	40 (20–97)	37 (23–87)	78 (24–173)	< 0.0001
Albumin (g L^−1^)	25 (20–29)	24 (20–29)	25 (20–29)	0.90
INR	1.4 (1.2–1.7)	1.4 (1.2–1.7)	1.5 (1.3–1.9)	< 0.001
Creatinine (μmol L^−1^)	95 (64–146)	77 (60–120)	134 (78–182)	< 0.000001
Platelet count (nL^−1^)	127 (81–181)	133 (83–181)	110 (70–162)	0.02
CYFRA21-1 (ng/mL)	5.37 (0.20–11.01)	5.21 (0.20–9.80)	8.94 (1.96–20.53)	< 0.0001

Quantitative measures are shown as median (interquartile range) or as an absolute count (*n*) and relative frequency (%). *Abbreviations*: *HCC* hepatocellular carcinoma, *SBP* spontaneous bacterial peritonitis, *MELD* model for end-stage liver disease, *ACLF* acute-on-chronic liver failure, *ALT* alanine aminotransferase, *AST* aspartate aminotransferase, *INR* international normalized ratio. *Including 38 patients who were lost to follow-up after a median of 14 days. **Non-survivors include patients who died (*n* = 78) or received a liver transplantation (*n* = 10) within 90 days after study recruitment

**Fig. 4:**
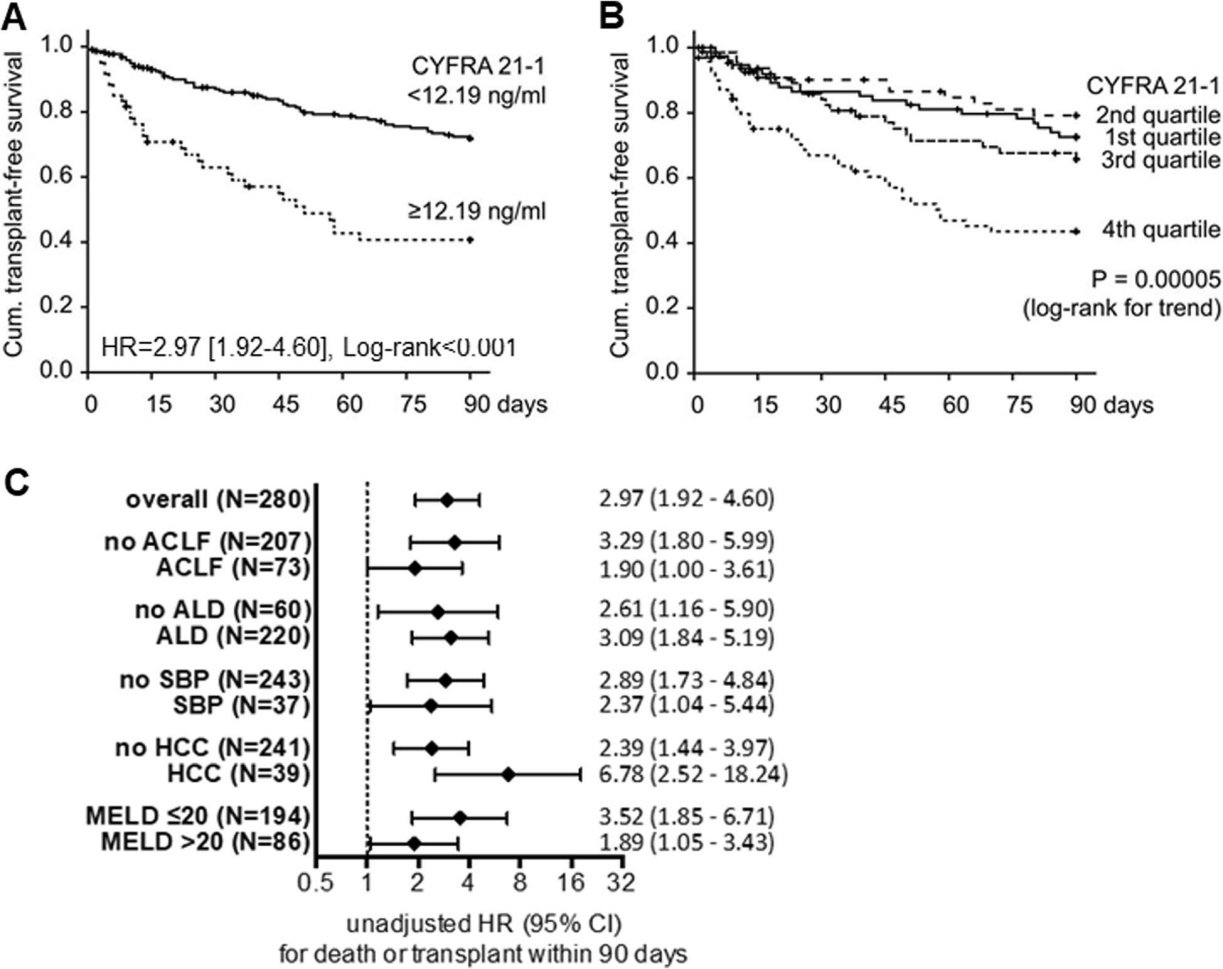
Serum CYFRA21-1 levels as an indicator of 90-day transplant-free survival in hospitalized patients with decompensated cirrhosis and ascites (cohort iv). Two hundred eighty patients who were hospitalized for decompensated cirrhosis and ascites were analyzed. Kaplan-Meier curves depict the cumulative 90-day transplant-free survival for serum CYFRA21-1 levels dichotomized at 12.19 ng/mL (**a**) and for quartiles of serum CYFRA21-1 concentrations (≤ 0.2 ng/mL, 0.2–5.4 ng/mL, 5.4–11.0 ng/mL, > 11.0 ng/mL) (**b**). Unadjusted hazard ratios (HRs) with their 95% confidence intervals [in brackets] and log-rank *P* values are shown in the corresponding graphs. **c** Sensitivity analysis of serum CYFRA21-1 for predicting 90-day transplant-free survival in indicated subgroups. Unadjusted hazard ratios (HRs) and 95% confidence intervals are displayed. Abbreviations: ACLF, acute-on-chronic liver failure; ALD, alcoholic liver disease; SBP, spontaneous bacterial peritonitis; HCC, hepatocellular carcinoma; MELD, model of end-stage liver disease

To estimate the diagnostic accuracy of serum CYFRA21-1 for 90-day transplant-free survival, we performed a ROC analysis. The AUROC for CYFRA 21-1 alone was 0.64 with an optimal cut-off being 12.19 ng/mL (sensitivity 36.4%; specificity 85.4%; Additional file 1: Figure S6). High serum CYFRA21-1 at the established cut-off indicated patients with a low 90-day transplant-free survival in the corresponding Kaplan-Meier curve analysis (Fig. 4a). Notably, CYFRA21-1 levels in the highest quartile conferred a particularly bad prognosis (Fig. 4b). While the composite scores MELD (AUROC 0.70) and ACLF grade (AUROC 0.68) performed numerically better than CYFRA 21-1 alone, the combination of MELD with CYFRA 21-1 numerically improved the AUROC to 0.73 (Additional file 1: Figure S6).

In multivariable Cox regression models, high serum CYFRA21-1 (unadjusted HR 2.97 [1.92–4.60]; *P* < .0001) remained a robust indicator of death or transplant within 90 days after adjustment for MELD, MELD and age, ACLF grade and age, and MELD and white blood cell count (WBC) (Additional file 1: Table S6). Similar results were obtained when CYFRA21-1 was assessed as a dichotomized and a continuous variable (Additional file 1: Table S6). Sensitivity analyses showed that the prognostic ability of serum CYFRA21-1 remained strong across several patient subgroups such as patients with/without ACLF, high/low MELD score, and with/without HCC (Fig. 4c).

Collectively, in patients with decompensated cirrhosis, serum CYFRA21-1 constituted an independent indicator of 90-day transplant-free survival (Table 4).

## Discussion

In our study, we evaluated the diagnostic relevance and prognostic significance of the K19-based CYFRA21-1 as a novel, direct serum marker of DR in different liver disease settings. We demonstrate that hepatic K19 expression remains unaltered in non-cirrhotic liver disease patients of various etiologies, which is in line with our previous findings [19]. In contrast, the expression of hepatocellular keratins K8/K18 rises already in intermediate fibrosis stages [19]. We also observed a strong correlation between the hepatic levels of K19 and the other DR keratins (K7/K23), thereby strongly suggesting that the detected elevated hepatic K19 expression mirrors an increased DR. Notably, DR is known to be particularly prominent in cirrhosis where regeneration through mitosis of mature hepatocytes becomes impaired [9, 10]. Future studies correlating the hepatic expression of K19 with the serum levels of CYFRA21-1 in a well-characterized cohort of patients with various stages of chronic liver disease (i.e., non-advanced and advanced) are warranted.

The major aim of our study was to evaluate the pathophysiologic associations underlying the prognostic implications of CYFRA21-1 levels. Notably, CYFRA21-1 displayed only weak correlations with the hepatocellular injury markers AST/ALT or M30/M65 and no/minimal correlation with liver synthesis parameters (Additional file 1: Table S5) suggesting that CYFRA21-1 mirrors liver disease severity rather than the synthetic ability of cirrhotic livers. This is in line with the current understanding of DR, that is thought to parallel liver disease severity [10, 11] as well as the studies employing immunohistochemical staining for ductular keratins, that also detected a correlation with liver disease severity [12, 17].

The extent of portal hypertension is an excellent indicator of liver disease severity and therefore has well-established prognostic implications [18]. Consequently, the predictive usefulness of CYFRA21-1 might be in part due to the fact that it correlates with the degree of portal hypertension. A correlation between DR and the extent of portal hypertension has been noted previously in a small series of patients with schistosomiasis (i.e., a cause of non-cirrhotic portal hypertension) undergoing direct portal pressure gradient [20] and is not surprising, since DR is known as the prevailing mode of regeneration in advanced/end-stage liver disease [10, 11]. However, portal hypertension due to schistosomiasis differs in several aspects from the etiologies of chronic liver diseases that were evaluated by HVPG measurement in our study.

Regarding prognostic implications, in all three cohorts of patients with ACLD (cohorts ii–iv), higher CYFRA21-1 values were associated with reduced survival. This association was independent of the underlying etiology, the disease severity (i.e., compensated or decompensated cirrhosis), and a variety of potentially relevant confounders (e.g., demographic parameters). Importantly, the associations also remained robustly significant after adjustment for the MELD score, Child-Pugh score, and ACLF grade, indicating its incremental benefit over classical scoring systems. In line with this clear and consistent association of serum CYFRA 21-1 with liver-related survival, multiple studies demonstrated the usefulness of histological DR quantification in the long-term prognosis of chronic liver disease. These typically relied on the immunohistochemical detection of ductular keratins K7/K19, as an indicator of the disease course [12–15]. Our findings extend these observations and demonstrate that DR products are useful not only as histological surrogates, but may also constitute attractive serum markers. This scenario is particularly attractive given that CYFRA21-1 is already widely available in the clinical routine, and thus, our findings are easy to implement.

Notably, elevated CYFRA21-1 levels also associated with both HCC occurrence and HCC-unrelated liver-related mortality. While the association with HCC occurrence was rather weak, K19 is abundantly produced in liver cancer stem cells and delineates a HCC subgroup with poor prognosis [21–23].

In all three cohorts of patients with ACLD, the median CYFRA21-1 serum levels were mostly above the upper limit of normal for the general population (e.g., median CYFRA21-1 levels in healthy controls were described as of 1.5 ng/mL [24]). While K19 is produced in several non-hepatic tissues [1, 25], these data suggest that the release of K19 from cirrhotic livers exceeds the K19 liberation from other organs. However, our study design cannot fully exclude the contribution of non-hepatic sources of K19.

CYFRA21-1 does not appear to have a broad diagnostic range as a majority of values were below 6 ng/mL. While continuous increases of serum CYFRA21-1 were significantly and independently associated with survival, the corresponding ORs and AUROCs were rather low (Additional file 1: Table 2, S4, and S6). Of note, using a cut-off determined by a cohort-specific Youden index (between 3.90 and 12.19 ng/mL), CYFRA21-1’s association with portal hypertension and survival was not only independent but also clear (i.e., ORs between 2 and 7). Given the steady hepatic K19 expression in non-cirrhotic fibrosis stages (cohort i) and the increasing relevance of DR as a primary source of regeneration with increasing parenchymal loss and the concomitant tissue remodeling [9, 10], CYFRA21-1 does not appear to be discriminative in non-advanced chronic liver disease. Instead, CYFRA21-1’s diagnostic utility lies rather in its association with CSPH and its independent survival prognostication when the serum levels are rather high. One might speculate that CYFRA21-1 is of particular prognostic relevance in decompensated cirrhosis and/or ACLF with a strong DR. However, further studies are warranted to determine CYFRA21-1’s utility in prospective cohorts of patients with advanced and non-advanced chronic liver disease.

## Conclusions

While CYFRA21-1 is a well-established and widely available serum marker of several non-hepatic tumors [5], our data strongly suggest that it can be repurposed to serve as the first direct serological marker of DR. Regardless of the underlying etiology, serum CYFRA21-1 levels are consistently elevated in different ACLD cohorts and are linked to the severity of portal hypertension. Furthermore, serum CYFRA21-1 levels appear to be a stable marker independent of conventional liver biochemistry markers and prognostic scores (e.g., MELD) pointing to an added benefit in terms of prognostication and pathophysiological insight. Importantly, CYFRA21-1 does not only reliably predict both the short- and long-term mortality of ACLD but also holds a great promise to promote our understanding of the biological importance of DR.

## Supplementary information

**Additional file 1.** Supplementary methods. Supplementary tables. Supplementary figures. References (Supplement).

## Data Availability

The datasets used and analyzed during the current study are available from the corresponding author on reasonable request.

## Abbreviations

ACLD: Advanced chronic liver disease
ACLF: Acute-on-chronic liver failure
CPS: Child-Pugh score
CSPH: Clinically significant portal hypertension
CYFRA21-1: Keratin 19 fragments/fragments of keratin 19
DR: Ductular reaction/ductal reaction
HCC: Hepatocellular carcinoma
HVPG: Hepatic venous pressure gradient
K19: Keratin 19
MELD: Model of end-stage liver disease
WBC: White blood cell count
