# Supplementary material for: Serum keratin 19 (CYFRA21-1) links ductular reaction with portal hypertension and outcome of various advanced liver diseases
Source: BMC Med. 2020 Nov 12;18:336. doi: 10.1186/s12916-020-01784-7 (PMC7661160; doi:10.1186/s12916-020-01784-7)
Supplement: Supplementary file 1 — Additional file 1. Supplementary methods. Supplementary tables. Supplementary figures. References (Supplement). [file 12916_2020_1784_MOESM1_ESM.docx]

**Online-only supplement of Hamesch et al. – “Serum keratin 19 (CYFRA21-1) links ductular reaction with portal hypertension and outcome of various advanced liver diseases.”**

Karim Hamesch, MD^1*^, Nurdan Guldiken, PhD^1*^, Mahmoud Aly, PhD^1,2^, Norbert Hüser, MD^3^, Daniel Hartmann, MD^3^, Pierre Rufat, MD^4^, Marianne Ziol, PhD^5,6,7^, Katharina Remih^1^, Georg Lurje, MD^8,9^, Bernhard Scheiner, MD^10^, Christian Trautwein, MD^1^, Mattias Mandorfer, MD, PhD^10^, Thomas Reiberger, MD^10^, Sebastian Mueller, MD, PhD^11^, Tony Bruns, MD^1,12,13^, Pierre Nahon, MD^14,15,16^, Pavel Strnad, MD^1#^

**TABLE OF CONTENTS**

Supplementary methods

Supplementary tables

Supplementary figures

References (Supplement)

# **SUPPLEMENTARY METHODS**

## **Analysis of human liver samples (Cohort i)**

Fifty seven liver samples from patients who underwent a liver biopsy for assessment of chronic liver disease at the University of Aachen, University of Ulm, and the Bondy Liver Unit between the years 2006-2018 were analyzed: alcoholic liver disease (ALD, n=13), chronic hepatitis C (HCV, n=17), non-alcoholic fatty liver disease (NAFLD, n=14). The diagnosis of HCV was confirmed by a positive HCV RNA test and all patients were treatment-naive. The diagnosis of ALD/NAFLD was based on laboratory parameters, histological findings, personal interview and an exclusion of other liver disease etiologies including viral infection, hereditary hemochromatosis, and autoimmune liver disease. ALD patients reported a daily alcohol consumption of >80 g in males and >60 g in females for at least 10 years. NAFLD patients had characteristic histological findings without significant alcohol consumption. 13 liver samples from surrounding, non-affected tissue of liver metastases from patients without known chronic liver disease had been collected at the University of Munich and were used as controls. Grading of inflammation and staging of fibrosis were done on 4µm-thick liver sections using Desmet (HCV, controls) and Kleiner scores (ALD, NAFLD).

All tissues were frozen immediately after biopsy or surgery. RNA was isolated from liver samples via RNeasy tissue fibrous tissue mini isolation kit (Qiagen, Hilden, Germany). The RNA samples were translated to cDNA with M-MLV reverse transcriptase kit (Promega, Madison, WI, USA). The relative expression of genes of interest was determined using specific primers for K19 (F: TAC AGC CAC TAC TAC ACG ACC ATC, R: AGA GCC TGT TCC GTC TCA AAC T), K7 (F: AGA CAT CTT TGA GGC CCA GAT, R: GGA CTG CAG CTC TGT CAA CTC), and K23 (F: GCC AGG ATG GCA GTG GAT GAC T, R: TCC TTC GGA GGC CCT CGA CT). The mRNA expression was adjusted based on the levels of the housekeeping ribosomal gene RPLPO (60S acidic ribosomal protein P0; F: GCA ATG TTG CCA GTG TCT GT, R: GCC TTG ACC TTT TCA GCA AG).

From the same liver samples, 3 control and 3 cirrhotic samples were chosen to examine K19 on protein level. Tissue lysates were prepared in homogenization buffer containing 3% sodium dodecyl sulphate (SDS) and were diluted with 4x reducing Laemmli buffer afterwards. Proteins were separated via 10% SDS–polyacrylamide gel electrophoresis and transferred to PVDF membranes (GE Healthcare/Amersham Biosciences, Germany) for immunoblotting. After incubation with the primary and horse radish peroxidase (HRP)-coupled secondary antibodies, the resulting HRP signal was detected with an ECL Detection kit (GE Healthcare/Amersham Biosciences, UK). Following antibodies were used: anti-K19 (BA17; Cell signalling, Leiden, Netherlands) and anti-tubulin (clone AA2; Sigma, Steinheim, Germany).

For immunohistochemistry, 3-4 µm thick liver sections were deparaffinized, rehydrated and a citrate-based antigen retrieval was performed (Vector H-3300, Vector Laboratories, Petersborough, UK) for 30 min, as recommended by the supplier. The immunodetection was carried out with a K19 (1:200 dilution; BA17, Cell signalling) primary and a biotin-conjugated secondary antibody (Vector BA-9200). The antibody conjugation step was followed by incubation with peroxidase-labelled streptavidin and the 3,3'-diaminobenzidine as a substrate (Vector SK4100).

Ethics approval was granted by the Institutional review board of Aachen university (EK 173/15). All patients provided written informed consent.

## **Cohort of patients with portal hypertension due to advanced chronic liver disease (cohort ii)**

Three hundred thirty three adults who have undergone invasive hepatic venous pressure gradient (HVPG) assessment at the Medical University of Vienna, Austria between August 2007 and December 2015 were analyzed. These patients have been reported previously, however, none of the previous studies included information on CYFRA21-1. HVPG measurements were performed in a standardized manner using a balloon catheter(S1) in the absence of medications with impact on the HVPG (e.g., non-selective beta blockers). All patients fulfilled the following inclusion criteria: (i) presence of portal hypertension due to advanced chronic liver disease (ACLD) as defined by a HVPG ≥6 mmHg, regardless of the underlying etiology. (ii) adults with a residence in Austria; (iii) CYFRA21-1 assessment at the time of HVPG measurement. Exclusion criteria were: (i) age <18 years and (ii) causes of non-cirrhotic portal hypertension.

Patients were followed after the time of HVPG measurement and characterized for the end points of hepatic decompensation (i.e., requirement of paracentesis, hospital admission for or development of grade 3/4 hepatic encephalopathy, and variceal bleeding) as well as liver-related death (i.e., deaths owing to complications of ACLD or HCC; liver transplantation was considered an event). Demographic, clinical, and laboratory data were extracted at baseline from electronic patient records.

Ethics approval was granted by “Ethikkommission der Medizinischen Universität Wien” (1526/2017). The requirement of informed consent was waived by the responsible ethics committee.

## **Cohort of patients with alcoholic cirrhosis included in HCC surveillance programs (cohort iii)**

Two hundred thirty-one adults with biopsy-proven alcoholic cirrhosis, who were consecutively referred to the Jean Verdier Hospital Liver Unit for diagnosis and management of alcoholic cirrhosis between January 2000 and January 2010, were enrolled. All patients were part of a HCC surveillance program and fulfilled the following inclusion criteria: (i) histologically proven cirrhosis as diagnosed by an experienced hepatopathologist; (ii) history of excessive alcohol consumption (>80 g per day in males and >60 g per day in females, both for at least 10 years); (iii) no evidence of relevant liver comorbidities (among others, no infection from HIV, HCV, or HBV); (iv) no evidence of HCC at the time of inclusion; (v) adults of Caucasian origin with a residence in France; (vi) availability of serum samples at the time of liver biopsy; (vii) written informed consent for use of serum samples and regular follow-ups. Exclusion criteria were the following: (i) age <18 years; (ii) pregnancy at the time of inclusion. For each patient, the date of inclusion equaled the date of the first liver biopsy showing cirrhosis. At the time of inclusion and serum sampling, patients were in a compensated state of cirrhosis. After inclusion, all patients were followed prospectively and evaluated at least every six months by physical examination and liver ultrasonography in the setting of HCC screening. If HCC was suspected, further workup was initiated.

The main endpoint was occurrence of HCC during follow-up. Follow-up ended at the date of death or liver transplantation (considered as liver-related death) or at the last recorded visit (or information gathering) within the 6 months prior to 31 December 2010. In the deceased patients, the likely cause of death was determined from the hospital files, by contacting patient relatives, or their general practitioner. All included patients were followed-up for at least two years. Patients lost during follow-up after this period were included in the analysis and censored at the date of the last recorded information. The median follow-up was 73 months. Further information is provided elsewhere (S2, S3).

The samples were provided by the CRB (liver disease biobank) Groupe Hospitalier Paris Seine-Saint-Denis BB-0033-00027 (“Comité d’Ethique d’Aulnay-Sous-Bois”). All patients provided written informed consent.

## **Cohort of hospitalized patients with decompensated cirrhosis and ascites (Cohort iv)**

Two hundred and eighty adults with cirrhosis, who were hospitalized with ascites and underwent paracentesis at the Jena University Hospital, Germany between October 2010 and June 2015, were analyzed. All patients fulfilled the following inclusion criteria: (i) presence of decompensated cirrhosis based on clinical, laboratory, endoscopic, and imaging criteria; (ii) presence of ascites due to portal hypertension; (iii) adults of Caucasian origin with a residence in Germany; (iv) availability of serum samples at the time of paracentesis; (v) written informed consent for use of frozen serum samples and regular follow-ups. Exclusion criteria were: (i) age <18 years; (ii) pregnancy at the time of inclusion; (iii) secondary causes of peritonitis (e.g., peritoneal carcinomatosis, postoperative peritonitis, or acute pancreatitis).

Patients were included at the time of paracentesis, which was performed according to European guidelines in all patients with new onset grade 2 or 3 ascites, worsening ascites, or any complication of cirrhosis and were prospectively characterized for the end points death from any cause and liver transplantation. Demographic and clinical data were collected at baseline. Chronic viral hepatitis, autoimmune, and metabolic liver diseases were assessed according to standard patient care. Unless mentioned otherwise, blood parameters were determined using routine laboratory analysis. Subsets of this cohort were reported in previously published studies (S4, S5). The EASL CLIF-C ACLF score was calculated to distinguish patients with acute-on-chronic liver failure (ACLF, grade 1-3) from those without ACLF (S6).

Ethics approval was granted by “Ethikkommission des Universitätsklinikums Jena“ 2880-08/10 and 3683-02/3. All patients provided written informed consent.

**Measurement of CYFRA 21-1 in patient sera**

For quantitative measurement of CYFRA 21-1, a commercially available enzyme-linked immunosorbent assay (ELISA) kit was used according to the manufacturer’s instructions (EIA-5070; working range: 0.15-50 ng/mL; DRG Instruments, Marburg, Germany).

**Ethics statements**

A written informed consent was obtained from all patients or their legal surrogates, if the requirement had not been waived by the local ethics committee. All sub-studies and sample analyses were conducted in compliance with the Declaration of Helsinki (Hong Kong amendment) as reflected in an approval by the local ethics committees of the participating centers (see above for further details).

# **SUPPLEMENTARY TABLES**

| Parameter | All  n=57 | F0  n=14 | F1  n=10 | F2  n=11 | F3  n=10 | F4  n=12 |
| --- | --- | --- | --- | --- | --- | --- |
| Age (years) | 54 (19.5) | 62.5 (24) | 51.5 (19.5) | 41 (24.0) | 52 (19.5) | 55 (13.3) |
| Women | 19 (33%) | 6 (31%) | 4 (21%) | 3 (16%) | 3 (16%) | 3 (16%) |
| Controls | 13 (23%) | 13 (100%) | - | - | - | - |
| ALD | 13 (23%) | - | - | 4 (31%) | 6 (46%) | 3 (23%) |
| NAFLD | 14 (24%) | - | 5 (36%) | 3 (21%) | 4 (29%) | 2 (14%) |
| HCV | 17 (30%) | 1 (6%) | 5 (29%) | 4 (24%) | - | 7 (41%) |

**Supplementary table S1. Characteristics of patients who provided liver specimen for molecular analysis (Cohort i).**

Quantitative measures are shown as median (interquartile range) or as an absolute count (n) and relative frequency (%). Abbreviations: ALD, alcoholic liver disease; NAFLD, non-alcoholic fatty liver disease; HCV, chronic hepatitis C.

|  | **Serum CYFRA21-1**  dichotomized ≥3.90 ng/mL | |
| --- | --- | --- |
|  | **Hazard ratio**  **(95% CI)** | ***P* value** |
| Unadjusted | 3.02 [1.78-5.13] | <0.001 |
| Adjusted for age and sex | 3.03 [1.78-5.15] | <0.001 |
| Adjusted for etiology | 2.89 [1.69-4.94] | <0.001 |
| Adjusted for age, sex, and etiology | 2.95 [1.72-5.05] | <0.001 |
| Adjusted for platelet count | 3.05 [1.79-5.18] | <0.001 |
| Adjusted for MELD score | 2.92 [1.68-5.07] | <0.001 |
| Adjusted for Child-Pugh points | 1.99 [1.07-3.69] | 0.030 |
| Adjusted for all above | 2.05[1.08-3.89] | 0.029 |

**Supplementary table S2. Cox regression models for liver-related death using dichotomized serum CYFRA21-1 values in patients with ACLD (Cohort ii).**

CYFRA21-1 was dichotomized using a cut-off determined by the maximum Youden index for the presence of clinically significant portal hypertension. Abbreviations: CYFRA21-1, fragments of keratin 19; MELD; model for end-stage liver disease.

|  | **CYFRA21-1**  **<5.26 ng/mL**  **n=115 (49.8%)** | **CYFRA21-1**  **≥5.26 ng/mL**  **n=116 (50.2%)** | ***P* value** |
| --- | --- | --- | --- |
| Age at cirrhosis diagnosis (years) | 55.8±1.0 | 57.3±0.9 | 0.11 |
| Male sex | 89 (77.3%) | 96 (82.7%) | 0.32 |
| BMI (kg/m^2^) | 27.3±0.6 | 27.8±0.5 | 0.28 |
| Diabetes mellitus | 36 (31.5%) | 34 (29.3%) | 0.77 |
| Ascites | 34 (29.5%) | 53 (45.6%) | 0.014 |
| Hepatic encephalopathy | 7 (6.1%) | 16 (13.7%) | 0.08 |
| Child-Pugh score (points) | 7.0±0.3 | 8.1±0.2 | 0.001 |
| HCC during follow-up | 25 (21.7) | 31 (26.7) | 0.40 |
| Death  *- HCC-related*  *- Liver-related*  *- Extra-hepatic* | 36 (36.7%)  *18 (50.0*%*)*  *17 (47.2*%*)*  *1 (2.7*%*)* | 62 (63.2%)  *26 (41.9*%*)*  *33 (53.2*%*)*  *3 (4.8*%*)* | 0.005  *0.08^°^* |
| ALT (x ULN) | 1.3±0.01 | 1.4±0.01 | 0.12 |
| AST (x ULN) | 2.0±0.2 | 2.2±0.1 | 0.07 |
| GGT (x ULN) | 6.0±0.5 | 6.2± 0.7 | 0.24 |
| Bilirubin (µmol/L) | 40.6±5.8 | 55.5±6.2 | 0.028 |
| Albumin (g/L) | 37.3±0.7 | 35.1±0.6 | 0.013 |
| Prothrombin level (% control) | 67.6±2.0 | 59.8±1.8 | 0.006 |
| Platelet count (10^3^/mm^3^) | 141.8±5.6 | 125.3±6.6 | 0.010 |

**Supplementary table S3. Characteristics of patient cohort with alcoholic cirrhosis as a function of median serum CYFRA21-1 levels (Cohort iii).**

231 patients with cirrhosis due to long-term alcohol misuse included in HCC surveillance programs are shown. All biological and clinical parameters were recorded at inclusion. Quantitative measures are shown as mean ± standard error of the mean or as an absolute count (n) and relative frequency (%). Liver transaminases are displayed as a multiple of the upper limit of normal (ULN). Abbreviations: BMI, body mass index; HCC, hepatocellular carcinoma; ALT, alanine aminotransferase; AST, aspartate aminotransferase; GGT, gamma-glutamyl transferase; PT, prothrombin time. ^°^ Comparison between the different causes of death in patients with low vs. high CYFRA21-1 levels. Patients who received a liver transplantation were interpreted as “liver-related death” or “HCC-related death”.

|  | **HCC** | | **Overall death** | |
| --- | --- | --- | --- | --- |
|  | **Cox univariate analysis** | **Cox multivariate analysis** | **Cox univariate analysis** | **Cox multivariate analysis** |
| **Age (per year)** | 1.06 [1.03-1.09] *P*<0.0001 | 1.04 [1.01-1.07]  *P*=0.007 | 1.03 [1.01-1.06]  *P*=0.001 | 1.04 [1.01-1.06]  *P*=0.002 |
| **Male gender** | 8.3 [1.9-33.3]  *P*=0.003 | 5.5 [1.3-22.7]  *P*=0.01 | NA | NA |
| **Child-Pugh score (per point)** | NA | NA | 1.19 [1.09-1.29]  *P*<0.0001 | 1.18 [1.06-1.32]  *P*=0.002 |
| **BMI (per kg/m^2^)** | 1.06 [1.01-1.11]  *P*=0.005 | 1.06 [1.008-1.11]  *P*=0.02 | NA | NA |
| **CYFRA21-1 (per ng/mL)** | NA | NA | 1.21 [1.06-1.38]  *P*=0.003 | 1.22 [1.02-1.43]  *P*=0.02 |

**Supplementary table S4. Features associated with the risk of hepatocellular carcinoma (HCC) occurrence and overall death (including transplantation) according to Cox’s proportional hazards model in patients with alcoholic cirrhosis (Cohort iii).**

The table displays the odds ratios with 95% confidence intervals in parentheses as well as the respective *P* values. Abbreviations: BMI, body mass index; NA, not applicable.

|  | **Serum CYFRA21-1** | |
| --- | --- | --- |
|  | **Non-parametric correlation** | ***P* value** |
| **Age** | 0.132 | 0.028 |
| **MELD** | 0.196 | 0.001 |
| **ACLF grade (0 to 3)** | 0.184 | 0.002 |
| **Creatinine** | 0.115 | 0.054 |
| **INR** | 0.079 | 0.19 |
| **Bilirubin** | 0.176 | 0.003 |
| **ALT** | 0.248 | 0.00004 |
| **AST** | 0.219 | 0.0009 |
| **Platelet count** | 0.028 | 0.64 |
| **White blood cells** | 0.141 | 0.018 |
| **C-reactive protein** | 0.121 | 0.045 |
| **Sodium** | 0.034 | 0.57 |
| **Albumin** | -0.046 | 0.45 |

**Supplementary table S5. Correlation of serum CYFRA21-1 with parameters of liver function and inflammation in hospitalized patients with decompensated cirrhosis and ascites (Cohort iv).**

The correlation values are based on Spearman’s rank correlation test. Abbreviations: CYFRA21-1, fragments of keratin 19; MELD, model for end-stage liver disease; INR, international normalized ratio; ALT, alanine aminotransferase; AST, aspartate aminotransferase; ACLF, acute-on-chronic liver failure.

|  | **Serum CYFRA21-1**  dichotomized ≥12.19 ng/mL | | **Serum CYFRA21-1**  per 1-log_e_ increase | |
| --- | --- | --- | --- | --- |
|  | **Hazard ratio**  **(95% CI)** | ***P* value** | **Hazard ratio**  **(95% CI)** | ***P* value** |
| Unadjusted | 2.97 (1.92-4.60) | <0.0001 | 1.26 (1.10-1.43) | 0.0005 |
| Adjusted for MELD | 2.13 (1.35-3.35) | 0.001 | 1.19 (1.05-1.35) | 0.006 |
| Adjusted for MELD and age* | 1.98 (1.26-3.10) | 0.003 | 1.17 (1.03-1.33) | 0.01 |
| Adjusted for ACLF and age* | 2.37 (1.52-3.68) | 0.0001 | 1.20 (1.05-1.36) | 0.007 |
| Adjusted for MELD and WBC* | 2.02 (1.27-3.20) | 0.003 | 1.17 (1.03-1.33) | 0.01 |

**Supplementary table S6. Cox Regression Models for 90-day transplant-free survival using dichotomized and continuous variables for serum CYFRA 21-1 in hospitalized patients with decompensated cirrhosis (Cohort iv).**

CYFRA 21-1 was dichotomized using a cut-off determined by the maximum Youden index. Abbreviations: CYFRA 21-1, fragments of keratin 19; MELD; model for end-stage liver disease; ACLF, acute-on-chronic liver failure; WBC, white blood cell count. * Variables were log_e_-normalized.

# **SUPPLEMENTARY FIGURES**


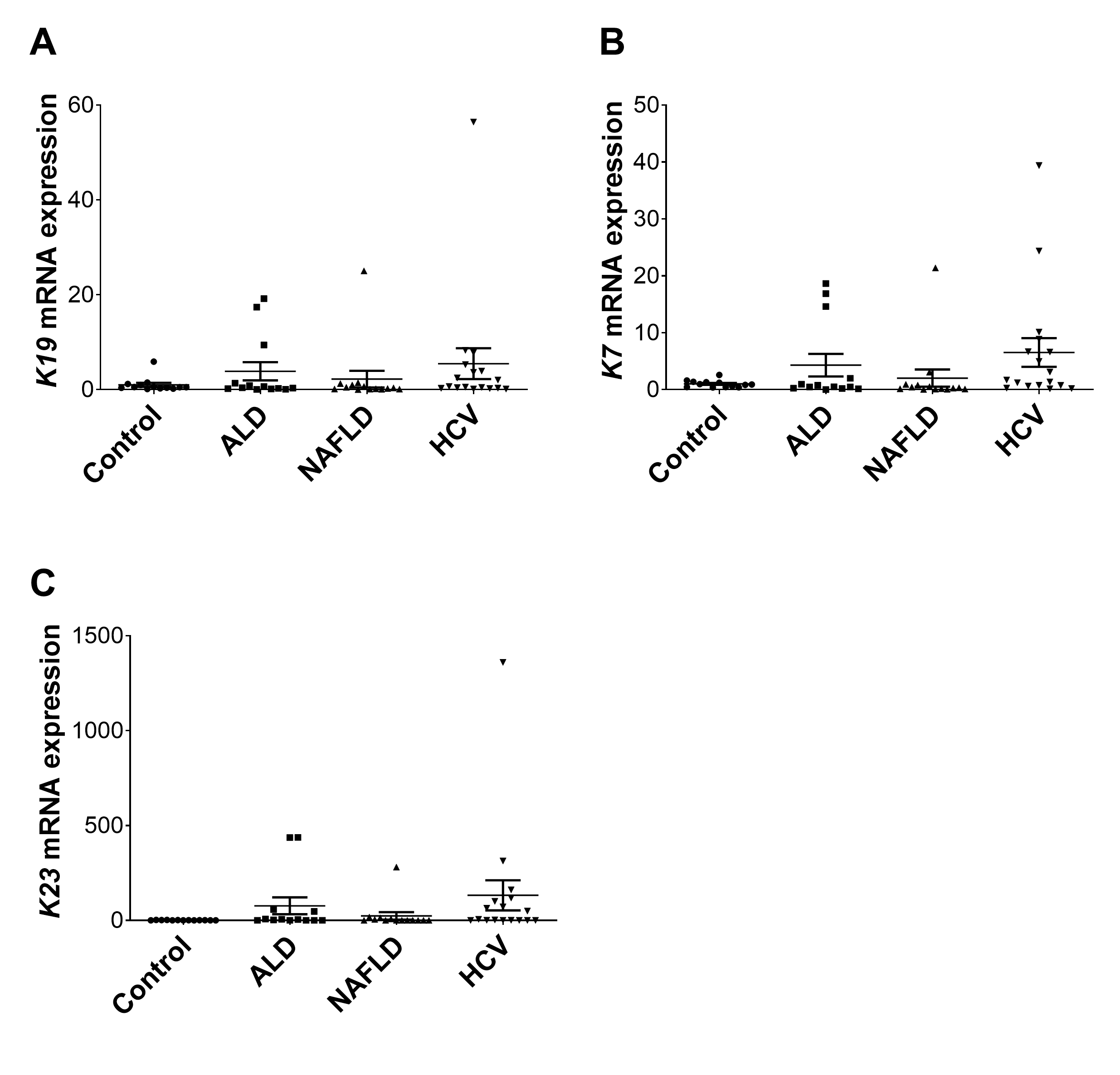


**Supplementary fig. S1. Expression of ductular keratins in liver samples of patients with and without chronic liver disease (Cohort i).**

Keratin 19 (K19; A), keratin 7 (K7; B) and keratin 23 (K23; C) mRNA levels were determined in control subjects as well as in patients with alcoholic/non-alcoholic fatty liver disease (ALD/NAFLD) and chronic hepatitis C infection (HCV). Average K7/19/23 expression in control subjects was arbitrarily set as 1 and all other levels represent a ratio. RPLP0 (ribosomal protein) gene was used as an internal control. There were no statistically significant differences between the respective groups.


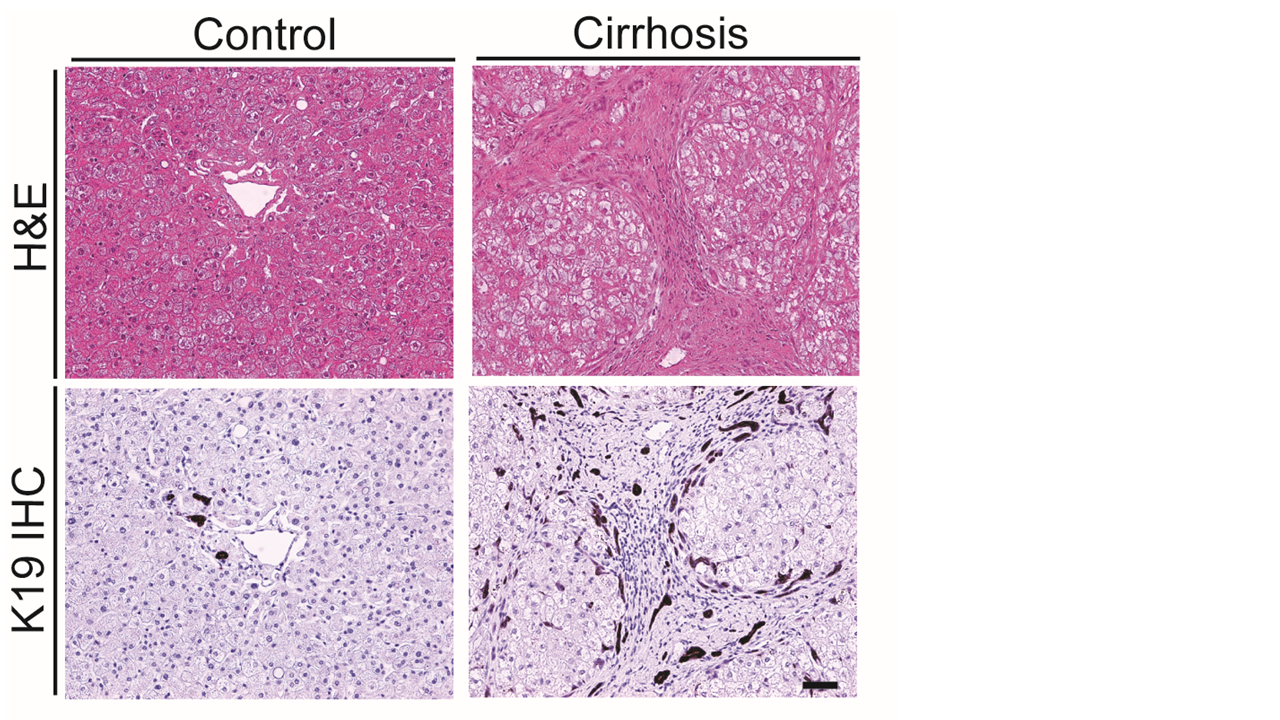


**Supplementary fig. S2. Hepatic keratin 19 (K19) levels and distribution in patients with and without cirrhosis (Cohort i).**

Representative liver sections stained with hematoxylin and eosin (H&E; upper panel) and with an antibody against K19 (lower panel). The left column displays sections from control patients without a significant liver disease (control), while the right column depicts sections from patients with hepatitis C-related cirrhosis (cirrhosis). Scale bars = 200 µm.


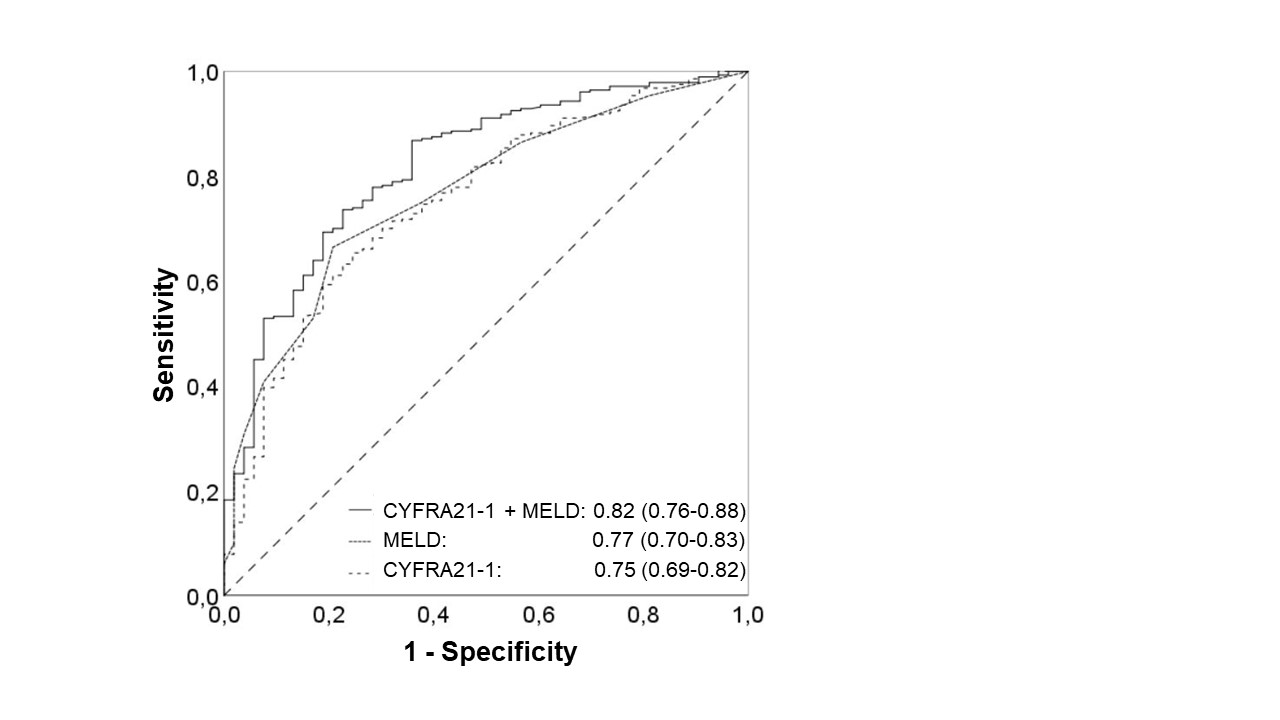


**Supplementary fig. S3. In patients with advanced chronic liver disease, serum CYFRA21-1 levels indicate the presence of clinically significant portal hypertension (CSPH) (Cohort ii).**

Receiver operating characteristics (ROC) curve for serum CYFRA 21-1 in relation to the presence of CSPH. Diagnostic accuracy for CYFRA21-1, model for end-stage liver disease (MELD) and the combination of MELD and CYFRA21-1 are shown. Areas under the ROC (AUROC) and the corresponding 95% confidence intervals are indicated.


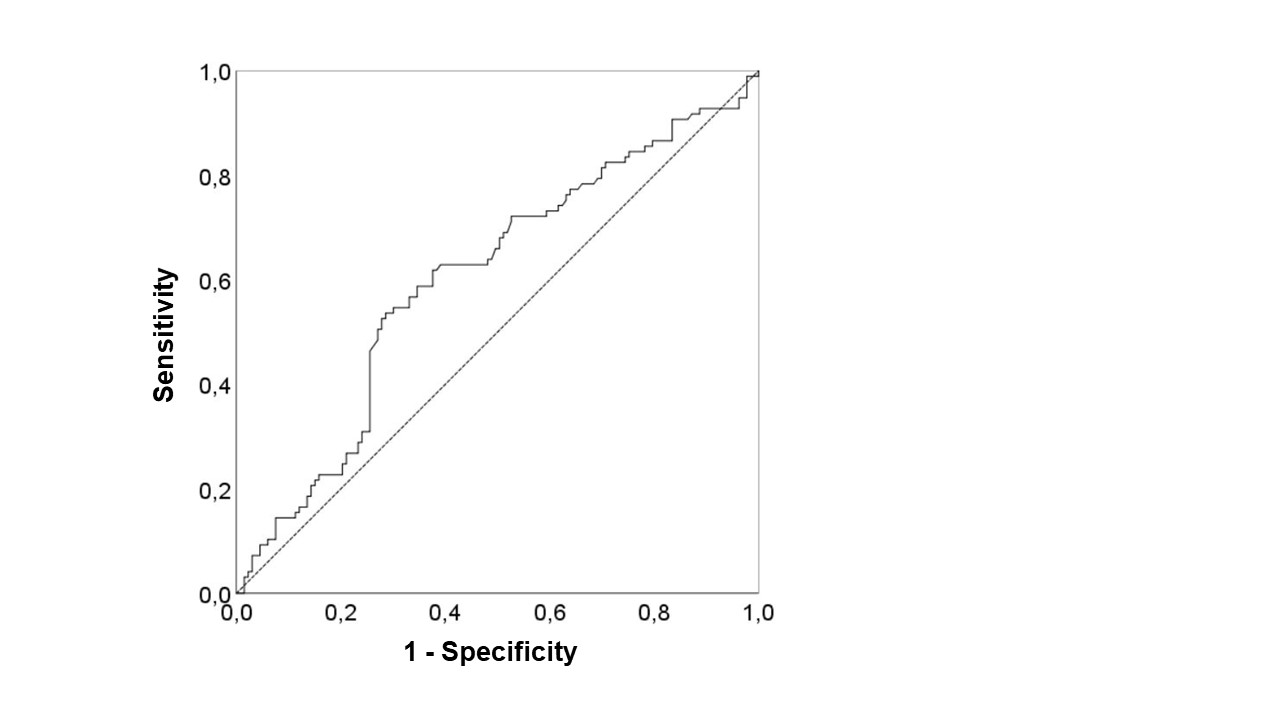


**Supplementary fig. S4. In patients with alcoholic cirrhosis, serum CYFRA21-1 levels indicate long-term survival (Cohort iii).**

Receiver operating characteristics (ROC) curve for serum CYFRA 21-1 in relation to long-term survival. Areas under the ROC (AUROC) and the corresponding 95% confidence intervals are indicated.


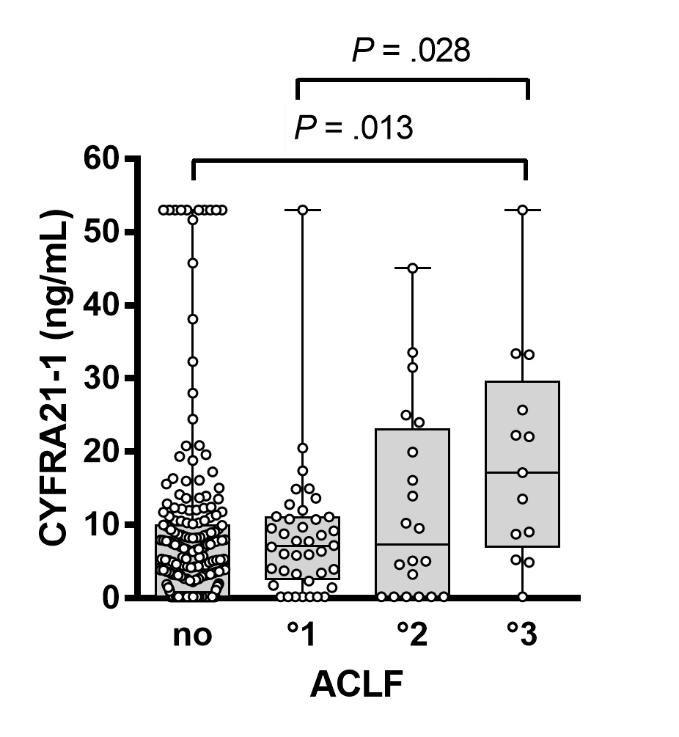


**Supplementary fig. S5. Serum CYFRA21-1 levels in hospitalized patients with acute-on-chronic liver failure (ACLF) (Cohort iv).**

Box plots in the four strata (no ACLF, ACLF grade 1, ACLF grade 2, and ACLF grade 3) are shown.


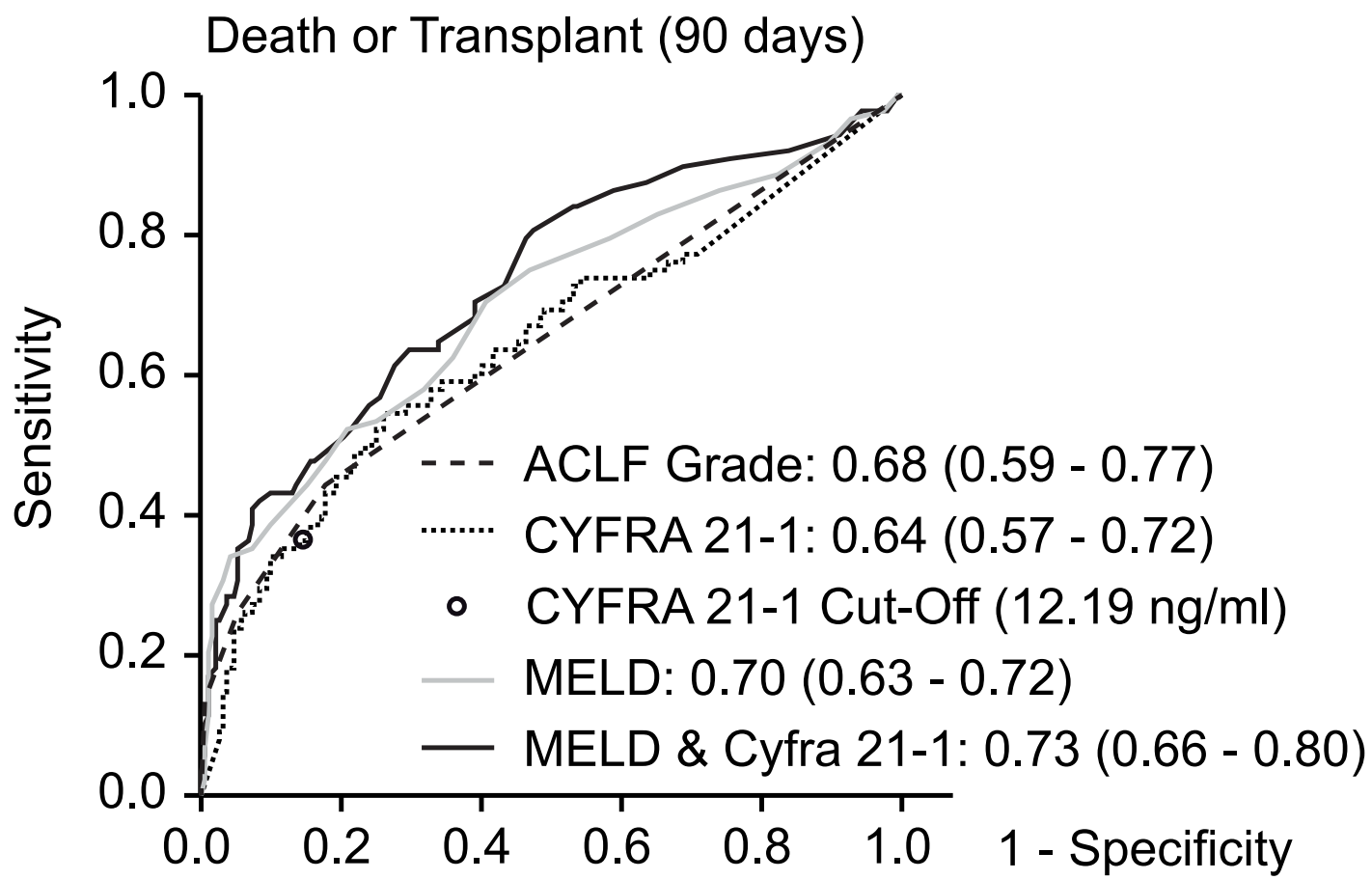


**Supplementary fig. S6. In hospitalized patients with decompensated cirrhosis and ascites, serum CYFRA21-1 levels indicate transplant-free survival (Cohort iv).**

Receiver operating characteristics (ROC) curve for serum CYFRA 21-1 in relation to 90-day transplant free survival. The optimal cut-off (sensitivity 36.4%, specificity 85.4%) corresponded to a serum CYFRA 21-1 level of 12.19 ng/dL. Diagnostic accuracy for CYFRA21-1, ACLF grade (0-3), model for end-stage liver disease (MELD) and the combination of MELD and CYFRA21-1 are shown. Areas under the ROC (AUROC) and the corresponding 95% confidence intervals are indicated.

# **REFERENCES (SUPPLEMENT)**

1. Ferlitsch A, Bota S, Paternostro R, Reiberger T, Mandorfer M, Heinisch B, et al. Evaluation of a new balloon occlusion catheter specifically designed for measurement of hepatic venous pressure gradient. Liver international : official journal of the International Association for the Study of the Liver. 2015;35(9):2115-20.

2. Nahon P, Nuraldeen R, Rufat P, Sutton A, Trautwein C, Strnad P. In alcoholic cirrhosis, low-serum hepcidin levels associate with poor long-term survival. Liver international : official journal of the International Association for the Study of the Liver. 2016;36(2):185-8.

3. Mueller S, Nahon P, Rausch V, Peccerella T, Silva I, Yagmur E, et al. Caspase-cleaved keratin-18 fragments increase during alcohol withdrawal and predict liver-related death in patients with alcoholic liver disease. Hepatology. 2017;66(1):96-107.

4. Bruns T, Nuraldeen R, Mai M, Stengel S, Zimmermann HW, Yagmur E, et al. Low serum transferrin correlates with acute-on-chronic organ failure and indicates short-term mortality in decompensated cirrhosis. Liver international : official journal of the International Association for the Study of the Liver. 2017;37(2):232-41.

5. Bruns T, Reuken PA, Stengel S, Gerber L, Appenrodt B, Schade JH, et al. The prognostic significance of bacterial DNA in patients with decompensated cirrhosis and suspected infection. Liver international : official journal of the International Association for the Study of the Liver. 2016;36(8):1133-42.

6. Moreau R, Jalan R, Gines P, Pavesi M, Angeli P, Cordoba J, et al. Acute-on-chronic liver failure is a distinct syndrome that develops in patients with acute decompensation of cirrhosis. Gastroenterology. 2013;144(7):1426-37, 37.e1-9.
